# 

**Published:** 1881-01-20

**Authors:** 


### TABLE OF CONTENTS.

ORIGINAL AND SELECTED ARTICLES.

Conversations upon the Physical and Mental Hygiene of Girlhood, by T. S. Powell, M.D, Atlanta, 1. Random Notes on Simplicity of Fracture-Apparatus, by William H. Morse, M.D, 9. Impotence, by Dr. Samuel W. Gross, 13. Break-Bone Fever, 15. Gastrotomy or Gastrostomy, by L. L. Staton, M.D, of N. C., 17.

ABSTRACTS AND GLEANINGS.

A New Knife for Fistula in Ano, 21. The Medical Year, 1880, 22. New Respirator; Fluid Extract of Ergot; Abnormally High Temperature; Desiccated, Defibrinated Blood, 23. Alkalies in Amenia; Chronic Dysentery and Diarrhœa; Camphor and Chloral Hydrate; Tonga; Carbonate of Ammonia; Electricity in Lead Colic, 24. Peritoneal Surgery; Intra-Uterine; Venesection; Jamaica Dogwood, 25. Chian Turpentine in Cancer; Otology; Boracic Acid in Surgery; Introduction of Tracheal Tubes by the Mouth; New Antiseptic; Quinine with Bromides, 26. Salicylate of Calcium; Ergot in Diabetes Mellitus; Sponge Tents; Pilocarpin, 27. Rheumatism, 28. For Chronic Rheumatism; Traumatic Tetanus, 29. Atrophy of Infants; Coca in the Opium Habit; Nasal Catarrh, 30.

SCIENTIFIC ITEMS.

The Prall System of Heating; Utilizing the Tide; Where our Forests are Going, 31. Population of the Earth; Transforming Sound into Light; Tenacity of Iron, 32.

PRACTICAL NOTES AND FORMULÆ.

Warner's Parvules; Tartaric Acid in Diphtheria; Petroleum Mass; Ergotine Solution for Hypodermic Use, 33. Removal of Spots, Stains, etc., 34. Cancer Cure; Amenorrhœa, Dysmenorrhœa, etc.; McMunn's Elixir, 35. Two Favorite Prescriptions; Asthma; Lent's Solution of Quinla; Burns, 36.

EDITORIAL AND MISCELLANEOUS.

Our Journal for 1881, 38. Book Notices, 39. Special Notices 40.

